# Language Outcomes in Adults with a History of Institutionalization: Behavioral and Neurophysiological Characterization

**DOI:** 10.1038/s41598-019-40007-9

**Published:** 2019-03-12

**Authors:** Sergey A. Kornilov, Marina A. Zhukova, Irina V. Ovchinnikova, Irina V. Golovanova, Oxana Yu. Naumova, Tatiana I. Logvinenko, Aleksandra O. Davydova, Maxim V. Petrov, Maria A. Chumakova, Elena L. Grigorenko

**Affiliations:** 1 Saint-Petersburg State University, Saint-Petersburg, Russian Federation; 2 Baylor College of Medicine, Houston, TX USA; 3 University of Houston, Houston, TX USA; 4 Vavilov Institute of General Genetics RAS, Moscow, Russian Federation; 5 National Research University Higher School of Economics, Moscow, Russian Federation; 6 Yale University, New Haven, CT USA; 7 Moscow State University for Psychology and Education, Moscow, Russian Federation

## Abstract

Impoverished early care environments are associated with developmental deficits in children raised in institutional settings. Despite the accumulation of evidence regarding deficits in general cognitive functioning in this population, less is known about the impact of institutionalization on language development at the level of brain and behavior. We examined language outcomes in young adults and adolescents raised in institutions (n = 23) as compared to their socioeconomic status and age peers raised in biological families (n = 24) using a behavioral language assessment and linguistic event-related potentials (ERPs). Controlling for intelligence, adults with a history of institutionalization demonstrated deficits in lexical and grammatical development and spelling. Analyses of ERP data revealed significant group differences in the dynamic processing of linguistic stimuli. Adults with a history of institutionalization displayed reduced neural sensitivity to violations of word expectancy, leading to reduced condition effects for temporo-spatial factors that tentatively corresponded to the N200, P300/N400, and phonological mismatch negativity. The results suggest that language is a vulnerable domain in adults with a history of institutionalization, the deficits in which are not explained by general developmental delays, and point to the pivotal role of early linguistic environment in the development of the neural networks involved in language processing.

## Introduction

It is estimated that over 2.7 million children in the world are raised in institutional settings because of the loss of both biological parents or their removal from the family due to other factors, such as the guardian’s inability to provide adequate care, protection, and education for the child^1^. Although alternative care systems such as foster care are gaining popularity, formal residential institutional care (IC) remains one of the most widely adopted systems for providing support for this vulnerable population in many countries, with only 1.4 million children worldwide estimated to be under an alternative (i.e., foster care) care system^1^. The increased attention to alternative care systems has been in part driven by the growing recognition of the negative effects of institutional care on children’s physical and psychological development due to the insufficient quality of early caregiving routinely provided in that environment. Evidence for the negative impact of institutionalization on child development should be interpreted cautiously in the context of the more global framework of diverse care environments across the world. Thus, although negative effects of institutionalization have been reported in a large number of studies in Eastern Europe and North America, efforts for global deinstitutionalization are challenged by recent evidence from low-income countries where institutional settings compare favorably to family-based care^2^. For example, in Sub-Saharan Africa, children raised in institutional settings are more likely to have their basic materials needs met when raised in institutional settings, compared to residential and community-based care^3^.

The Bucharest Early Intervention Project, BEIP^4,5^ drew international attention to the negative effects of institutionalization by documenting substantial developmental delays in children raised in Romanian orphanages, where the institutional care environment was characterized by severe physical and psychosocial deprivation and neglect. This and other studies indicated that children who had experienced early IC showed pronounced delays in physical growth^6^, deficits in motor development^7^, and profound delays in cognitive development and functioning^8–10^. A meta-analysis of 75 studies indicated that children in IC lag behind their peers raised in biological families, with a large effect size of *d* = 0.74^11^.

Deficits in the general cognitive functioning of children with a history of IC are hypothesized to be related to the complex pattern of atypicalities in brain development documented in this vulnerable population. Both gray and white matter deficits have been noted, ranging from lower cortical volume^12,13^ to atypical white matter structure in the prefrontal cortex, corpus callosum, and uncinate fasciculus^14–16^. Functional neuroimaging studies also revealed that individuals with a history of IC display atypical patterns of electrical brain activity at rest, with the EEG power spectrum distribution pointing to significantly delayed cortical maturation^17,18^.

In addition to revealing general cognitive deficits and deficits in socio-emotional functioning in children with a history of IC, studies have demonstrated that they show substantial delays in language development, compared to children who were placed in foster care and the never institutionalized group^19^, in some cases resulting in the absence of coherent functional language output at the age of 30 months. The follow-up study of BEIP participants at 8 years indicated that children assigned to foster care were able to utter longer and more complex sentences, displayed better written word identification and non-word repetition skills than children who remained in institutions, but had lower grammatical abilities than their never institutionalized age peers. Poor language outcomes have also been reported in a recent study of children raised in IC in Greece^20^. Although in one study of children adopted from Eastern Europe to the USA, age at adoption was unrelated to language outcomes beyond the age of three^21^, a five-year longitudinal study investigating the effects of different types of placement on children’s language outcomes suggested that, regardless of placement type, children exposed to early adversity remained below the population mean with respect to their language functioning^22^. Croft and colleagues^23^ and Loman and colleagues^10^ found that children with a history of institutionalization (even if it ended before the age of 6 months) consistently underperformed, when 6–11 years of age, on tests of spoken and written language development compared to the never institutionalized children and children placed into foster care in early infancy. Eigsti and colleagues^24^ demonstrated, in a sample of internationally adopted children aged 4–13 years, that top-down cognitive assessments including measures of explicit memory and cognitive control differed between children who experienced early IC and matched children from biological families, yet differences between the groups in bottom-up implicit learning processes were unremarkable. Thus, although the results are complex, they have important implications for our understanding of the unique needs of this population given the critical role of language as a higher-order ability that underlies communication, learning, and self-regulation, and its strong predictive power with respect to outcomes in a variety of domains for typically developing and vulnerable populations^25–28^.

The scarcity of research on language development in children raised in IC currently does not allow us to pinpoint the exact cause of these deficits. Nonetheless, the profile of these deficits is consistent with several possible explanations. First, given the documented general cognitive deficits in this population, it is possible that language suffers due to deficits in general learning mechanisms that support language development. One such mechanism is statistical learning, i.e., a hypothesized machinery for language learning that supports such processes as speech segmentation and the identification of word boundaries^29,30^ as well as organizing individual words into grammatical categories and learning the relationships between them^31,32^. Although the robust measurement of individual differences in statistical learning as the ability to extract stochastic properties of incoming linguistic input poses unique methodological and theoretical challenges^33^, and longitudinal data are lacking^34^, studies suggest that statistical learning is indeed predictive of language outcomes in children as well as of learning outcomes on a set of artificial grammar tasks for adults^35,36^. It is therefore possible that immature or impaired learning in children with IC launches a cascade of developmental deficits that have long-term effects on language development.

Second, it is possible that these mechanisms are intact (or even mildly affected) yet the child’s early linguistic environment in IC is insufficient for proper language development, due to the low quantity or quality of linguistic input and child-caregiver interactions^19,37,38^. In normative samples, children’s language development has been reported to be predicted by the amount of received linguistic input^39–41^ as well as by social context, e.g., infant-directed speech that occurs during joint attention and parental speech style, e.g., parentese^42^. In a recent study by Rowe, Leech, and Cabrera^43^, children’s language development was related to father’s use of *wh*-questions, a challenging type of input that prompted more complex responses on behalf of the child. For internationally adopted children, their delayed exposure is thought to be related to difficulties in the acquisition of grammar in the adopted language^44^. Together, these findings point to contextually-driven linguistic input as a potentially vulnerable element of the early care environment in formal IC.

Thus, studies of children with a history of IC suggest that, in addition to displaying general cognitive deficits, children raised in conditions with insufficient linguistic input display delays in the acquisition of their native language. Given the presence of sensitive and critical periods in language development and its dependence on a cascade of developmental processes in different domains, it is likely that early delays in language development might persist into young adolescence and adulthood. Language plasticity assumes a certain degree of compensation, evident in studies that examined adoptees and children placed in foster care. Yet, there is currently little published data on long-term language outcomes in post-institutionalized adolescents and adults. In one study, adolescents with a history of IC that were adopted before the age of 4 showed profound speech and language problems at the age of 11 years^45^. Another study found that age at adoption is an important factor for future language development, and length of institutionalization was the single most robust predictor of language outcomes^46^.

This brief review of the literature points to several gaps in our current understanding of the cognitive vulnerabilities in children raised in IC. Despite the fact that the presence of language deficits in children with a history of institutionalization is well-established, long-term language outcomes in adolescence and adulthood have not been systematically investigated with respect to both the identification of particularly vulnerable language domains and the characterization of the neurophysiological bases of language processing in this special vulnerable population. The latter is especially important given that compensation can result in end-point performance similar to that produced by an initially optimally developing system by implicating other neurocognitive systems. Thus, the aims of the empirical study reported in this manuscript were:

1. To perform a comprehensive evaluation of language skills in adults with and without a history of IC. We anticipated group differences between individuals with and without IC; yet, the manigitude of these differences needed to be empirically estimated.
2. To characterize language processing at the neural level by evaluating patterns of event-related brain activity using high-density EEG recordings in adults with and without a history of IC. We expected to see reduced amplitude in the components in the IC group that index the efficiency of language processing.

## Methods

### Participants, recruitment, procedure

Participants for the current study were recruited through a number of vocational schools located in a large city in the Russian Federation. We recruited participants specifically through schools that support vulnerable student populations from disadvantaged backgrounds, including adolescents and young adults who were left without biological parental care and were raised in institutional settings (baby homes and orphanages).

A total of 59 adults were recruited into the study. However, the analyses reported in this manuscript are based on the reduced sample of n = 47 individuals, following the application of strict inclusion/exclusion criteria: nonverbal intelligence within the normal range (i.e., standardized IQ score between 70 and 130 points); absence of uncorrected hearing or sight problems; no diagnosed neurological disorder (e.g., epilepsy, cerebral palsy) or neurological symptoms; or failure to provide at least 15 trials per each of the experimental condition. All participants reported Russian as their native language.

The final group composition was as follows. The group of individuals with a history of early institutionalization (Institutional Care, IC) was comprised of n = 23 adolescents and adults in the age range from 16.42 to 37.58 years (*M* = 22.05, *SD* = 6.20; 18 males), with the median reported duration of institutionalization of 10 years, and who lived in a median number of 2 institutions (baby homes and orphanages). Five individuals in the IC group also reported being placed at some point in a foster family or adopted. The comparison group of adults raised by their biological families (biological family care, BFC) was comprised of n = 24 adolescents and adults in the age range from 16.75 to 31.25 years (*M* = 22.89, *SD* = 4.84; 14 males). Importantly, the control group of their age matched peers raised by their biological families, was also primarily recruited from the same colleges. Although the groups did not statistically significantly differ on age, *t*_(45)_ = −0.51, *p* = 0.610, Cohen’s *d* = −0.15, or gender distribution, *X*^2^_(1)_ = 2.15, *p* = 0.143, both demographic variables were included (along with nonverbal intelligence, see below) as covariates in all of the reported analyses.

The participants completed a set of behavioral assessments (along with a self-reported medical history), followed by the acquisition of an EEG during a set of psycholinguistic tasks.

All participants provided oral and written informed consent. Consent for participation was obtained from the vocational school officials for individuals above the age of 16 and below the age of 18. Participation in the study was compensated with gift cards. All data collection procedures, study materials and informed consent forms were approved by the Internal Review Boards (Ethical Committee) of Saint Petersburg State University, and all study procedures and methods were carried out in accordance with these protocols.

### Behavioral assessment

#### Nonverbal intelligence

Given the current deficit of standardized measures of nonverbal intelligence in Russian and our successful experience in utilizing this measure in a variety of research projects in Russia^47^, we chose the Culture-Fair Intelligence Test (CFIT), Scale 2^48^ to measure nonverbal intelligence. The test contains four timed subtests (Matrices, Series, Classification, and Conditions) with figural material that evaluate the individual’s fluid or nonverbal intelligence in a paper and pencil format. As the test is not normed on the Russian population, we used the norms obtained for US adolescents and adults reported in the test manual for the purposes of excluding individuals who performed at the extremes of the nonverbal cognitive functioning continuum, and for the purposes of obtaining descriptive statistics. Raw scores were used in all subsequent analyses (the results from the analyses with raw vs. scaled scores were predictably identical given that all of the study participants fall in the same age band in the norming tables in the CFIT manual).

#### Language development

To evaluate language development and functioning, we used a recently developed comprehensive assessment of spoken and written Russian language called *АРФА-РУС* or, in the English transliteration, ARFA^49^. ARFA was developed to address the lack of standardized, well-discriminating measures of language development in adults in general and in Russian-speaking adults in particular. It includes seven subtests targeting various language domains. In Verbal Analogies (Cronbach’s α = 0.71), participants are asked to judge the similarity of meaning of pairs of words. In Word Definitions (Cronbach’s α = 0.79), participants are given a word in a sentential context and asked to provide a definition for it. In Sentence Comprehension (Cronbach’s α = 0.89), participants carry out actions with plastic tokens following controlled syntactically complex instructions. In Sentence Repetition (Cronbach’s α = 0.93), participants are asked to repeat sentences of varying length and syntactic complexity. The Pseudoword Repetition (Cronbach’s α = 0.80) and Phoneme Awareness (Cronbach’s α = 0.82) subtests assess participants’ phonological short-term memory, and their ability to manipulate novel phonological strings. Finally, a Spelling task (Cronbach’s α = 0.90) was used to probe literacy development, as spelling has been shown to be one of the most sensitive predictors of reading difficulties in Russian. The subtests and items were designed to follow the gold standards of language assessment development, and were largely evidence-based, modeling procedures previously shown to be particularly sensitive to language difficulties in adults. The instrument is available from the authors upon request.

### Experimental stimuli and procedure

For the purpose of this study, we adapted the cross-modal picture-word matching paradigm to create an ERP task that would probe participants’ lexical processing. A similar paradigm has been previously successfully used with developmental populations^50^, as well as populations with developmental disorders^47^, including adolescents and adults with language and reading deficits^51^. In this paradigm, participants are presented with pictures of objects and a set of spoken words; they are then asked to judge whether the word matched the object on the screen.

The experiment consisted of 180 trials, distributed across four conditions. In the match condition (90 trials; 30 picture-word pairs, each of which was repeated 3 times), the presented word matched the object in the picture (e.g., hear TORT “cake” – see tort “cake”). The match condition served as the baseline; for each picture-word pair data were averaged across the 3 repetitions. There were also three mismatch conditions: 1) in the initial phonological overlap condition (IPO, 30 trials), the word heard matched on the initial phoneme of the word represented by the object (e.g., hear TORS “trunk” – see tort “cake”); 2) in the semantically associated (SA, 30 trials) condition, the word did not overlap with the object phonologically but instead was semantically associated with it (e.g., hear CHAI “tea” – see tort “cake”); and 3) in the phonologically and semantically unrelated (UR, 30 trials) condition, the word was not related to the object, neither phonologically nor semantically (e.g., hear SAT “garden” – see tort “cake”). The order of trials was randomized across participants. Thirty images were selected from a commercial stock photo database; there were 120 words that were used in combination with these. Stimuli presentation was controlled by E-Prime 2.0 software (Psychology Service Tools, Inc).

At the beginning of each trial, a fixation cross is presented on the screen for 250 ms. Directly after that, the fixation cross is replaced by a picture for a 1000 ms preview period. Then, a spoken word is presented binaurally through open field speakers. The picture remains on the screen until the participant has pressed “1” (MATCH) or “2” (NO MATCH) on the Chronos response device (Psychology Service Tools, Inc), after which a new trial begins.

Prior to testing, all participants were familiarized with the procedure. Highly imageable and frequent words were selected from the frequency dictionary of Russian language^52^. The words across the four experimental conditions did not differ in either frequency or phonemic length (*p*’s > 0.05). The words were recorded by an adult female native Russian speaker using PRAAT audio software^53^ at a sample rate of 44100 Hz, and presented at 70 dB (SPL) using a set of Yamaha NS-BP300 speakers.

### EEG signal acquisition

The EEG signal was acquired using a high-density EEG system via a PC laptop running PyCorder software (BrainProducts, Inc). Specifically, we used the actiCHamp amplifier (BrainProducts, Inc) to record EEG from the scalp using 64 Ag/AgCl sintered active electrodes mounted in an elastic cap according to the standard 10–10 montage using SuperVisc electrolyte gel. The signal was recorded using linked mastoids as the reference and digitized at 1000 Hz. All impedances were kept below 25 kΩ. The electrooculogram was monitored using the bipolar vertical and horizontal EOG electrodes. Data from these electrodes and from the frontal electrodes Fp1 and Fp2 were excluded from the analysis following the ICA-based correction of eye movement-induced artifacts.

### Data analysis

#### Behavioral data scoring and general inferential statistics

All analyses were performed in Microsoft’s (Microsoft, Inc) OpenMP implementation of the R programming environment v 3.4.2 running on a Linux server (governed by the RStudio Server software).

For the behavioral assessments, the resulting scores represented the summed scores for subtest items. In order to obtain, in addition to subtest-level indicators, an index of general language development, we extracted the first unrotated principal component from the ARFA scores using the *prcomp*() function. To simplify visual comparisons and the heuristic interpretation of effect sizes, the ARFA scores for both groups were standardized using the mean of the BFC group and the pooled standard deviation, then scores were re-scaled to have a mean of 100 and SD of 15. CFIT scores remained scaled according to the manual’s norms.

Given that developmental and clinical populations are frequently characterized by high variability in scores and the presence of outliers, accurate inference requires special care to account for these characteristics. Correspondingly, we used a set of so-called robust methods for estimation. Group differences in the behavioral indices of language and cognitive development were modeled by fitting a set of robust linear models as implemented in the *lmrob*() function (package robustbase)^54^. This function computes a two-step MM-type regression estimator^55,56^, which finds an S-estimate that minimizes the M-estimates of the residual scales. Holding the estimated scale constant, a close-by M-estimate of the parameters is then located. This method provides robustness to outlying or highly-influential data points. Parameter estimates are otherwise interpreted similarly to standard parameters from the general linear model class. Continuous covariates (age, intelligence) were mean-centered prior to the analysis.

Group differences in behavioral performance were examined using a set of 2 (Group: IC, BFC) X 4 (Condition: Target, IPO, SA, UR) mixed logit models fitted to the item-level data with crossed random effects for items (trials) and subjects. Mixed effects logit models are a type of generalized linear mixed model that employs a logit link function for dichotomous dependent variables. In these models, the outcome is analyzed as the linear combination of fixed (corresponding to the effects of independent variables) and random effects (e.g., participant- and item-specific effects that model variability at the level of individual participants and items, respectively). This approach is particularly suitable for analyzing item-level binary accuracy data and has important advantages^57–59^ over traditional ANOVA performed on average accuracy scores: e.g., mixed models do not make the assumption of homogeneity of variance, and have overall higher statistical power combined with better control of Type I error. The modeling of conditional response probabilities was performed using the *lmer()* function from the *lme4* library^60^ with Laplace approximation. The target condition, BFC group, and female gender were used as reference levels in this regression-based approach.

Group differences in log-transformed reaction times and ICA-based measures of event-related potentials (see below) were also modeled using the robust implementation of mixed linear models from the *robustlmm* library^61^. The use of *p*-value estimation for fixed effects in mixed models is controversial, particularly due to the difficulty of estimating the number of degrees of freedom for these models. To the best of our knowledge, robust implementations of such solutions as the Sutterwoth approximation utilized in the *lmerTest* library for non-robust mixed models are currently lacking, and confidence intervals produced by bootstrapping are not robust in these cases. Correspondingly, we decided to follow the t-as-z approach to heuristically evaluate the significance of the fixed effects. Although the general rule of thumb is to evaluate the *p*-values using the *t*-value statistic threshold of |2| as the cut-off for determining the statistical significance of the effect (at two-tailed *p* < 0.05)^62^, we adopted a more stringent threshold for the t-value statistic of |2.326|, roughly corresponding to a two-tailed significance level of *p* < 0.01. Throughout the text, we provide B effect estimates and their standard errors (SEs) for all fixed effects.

We also performed quadratic discriminant analysis (QDA) using the *qda()* function from the MASS library^63^ with a robust t-estimator. QDA is similar to linear discriminant analysis (and logistic regression) and also assumes that the predictor variables are drawn from a multivariate Gaussian distribution but relaxes the constraint on the equality of covariance structures between the two classes. Posterior probabilities were used to establish class (i.e., group) membership. The ROCR library^64^ was used for receiver operating curve (ROC) plotting. Correlational analyses were performed using Spearman’s rho coefficient.

#### EEG signal processing and temporo-spatial independent component analysis (ICA)

The data were processed off-line using BrainVision Analyzer software v 2.1 (BrainProducts, Inc). At the first step, the signal was downsampled to 500 Hz. A small number of channels were reconstructed using spheric spline interpolation if deemed necessary based on the visual inspection of the full data set for each participant. We then re-referenced the signal to the common average reference. We then applied an IIR bandpass (0.10 to 35 Hz) filter to the signal, followed by a 50 Hz notch filter to selectively attenuate power line noise. The EEG signal was segmented into 900 ms epochs with a 200 ms pre-stimulus baseline. Following local DC detrending applied to the extracted segments, we used the independent components analysis (ICA) based ocular artifact correction procedure; performance of the ICA algorithm was controlled manually by the inspection of suggested ocular components with prominent frontal topography and peak power in the EOG electrodes.

We only analyzed segments with correct behavioral responses that passed our artifact rejection criteria: a voltage step of no more than 50 uV in the segment; an absolute voltage that does not exceed ±150 uV in any of the EEG channels. The segments were averaged separately for each of the four conditions. The average number of trials included in the analysis did not differ significantly between the two groups in all of the conditions (all *p*’s > 0.09).

The data were then exported to EP ToolKit^65,66^ where two-step temporo-spatial independent component analysis (ICA) was performed on subject-level averaged waveforms for all conditions simultaneously. Temporo-spatial ICA is a two-step procedure that capitalizes on the availability of high-density EEG data and high temporal resolution EEG to identify consistent patterns (“components”) in the EEG (including event-related) activity. This method is an alternative to conventional approaches that rely on the visual identification of electrode clusters and time windows for peak extraction and latency measurement. These were data-driven attempts to separate the waveforms within a set of overlapping components and sub-components, each characterized by a temporal as well as spatial configuration. In temporo-spatial PCA, initial temporal ICA scores from the analysis that identify components with distinct time courses are then subjected to spatial ICA in an attempt to further unconfound the components by identifying topographic patterns in the spatial domain. The selection of the number of retained factors (or components) is guided by the parallel scree test that compares the eigenvalues obtained from the data to those obtained using simulated random data. Each resulting component represents a temporo-spatial factor, and can be quantified (in terms of its “amplitude” or prominence) with a factor score, then described in terms of its peak latency and peak channel (as well as a topographic vector).

Coupled with guided expectations rooted in theory and prior knowledge regarding the paradigm and the components of interest, this method provides stable, interpretable results in basic as well as clinical and applied studies. Moreover, as we have previously shown, results obtained with temporo-spatial ICA components not only closely correspond to (and correlate with) those obtained through amplitude and latency modeling of visually identified components, but actually increase statistical power^67^.

## Results

### Cognitive and language outcomes in adults with a history of institutionalization

In this group comparison, we anticipated a consistent depressed performance of adults with a history of institutionalization on all of the administered tasks. To establish the profile of language performance in adults with a history of institutionalization, three sets of analyses were performed. First, we compared the two groups on nonverbal intelligence. Second, we examined group differences in participants’ performance on the ARFA subtests. Finally, we examined their behavioral performance (accuracy and reaction time) in the cross-modal picture-word matching paradigm.

Adults with a history of early institutionalization displayed significantly lower nonverbal intelligence scores (Institutional Care, IC, group: *M* = 90.61, *SD* = 10.21) than adults raised by their biological families (Biological Family Care, BFC, group: *M* = 102.92, *SD* = 13.80; covarying for age and sex, regression *B* = −13.70, *SE* = 3.65, *t* = −3.79, *p* = 0.0005). This effect size is consistent with prior literature documenting pronounced cognitive development delays in children raised in impoverished environments, including children raised in orphanages^11,68^. In keeping with the goal of the study, we therefore made the decision to include nonverbal intelligence as a mean-centered covariate in all of the subsequent analyses reported in this manuscript.

**Figure 1:**
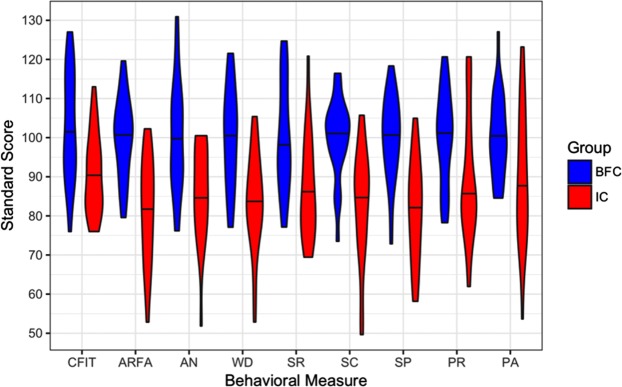
Violin plots of behavioral assessment scores in two groups of participants – adolescents and adults with a history of institutionalization (IC) and their age peers raised by biological families (BFC). Horizontal lines represent median group scores. CFIT – Cattell’s Culture Fair Intelligence Test, ARFA – Assessment of the Development of Russian (PCA composite), AN – Analogies, WD – Word Definitions, SR – Sentence Repetition, SC – Sentence Comprehension, SP – Spelling, PR – Pseudoword Repetition, PA – Phoneme Awareness.

Controlling for age, sex, and nonverbal intelligence, adults with a history of IC demonstrated a depressed performance profile across ARFA subtests (see Fig. 1), including Analogies (*B* = −10.08, *SE* = 4.31, *t* = −2.34, *p* = 0.0243), Word Definitions (*B* = −10.05, *SE* = 3.91, *t* = −2.57, *p* = 0.0138), Sentence Comprehension (*B* = −8.92, *SE* = 3.53, *t* = −2.53, *p* = 0.0153), Sentence Repetition (*B* = −8.84, *SE* = 4.12, *t* = −2.15, *p* = 0.0377), with the largest effect obtained for Spelling (*B* = −15.92, *SE* = 4.38, *t* = −3.63, *p* = 0.0007). Although nonverbal intelligence positively predicted the general language development score obtained through the principal component analysis (*B* = 0.46, *SE* = 0.14, *t* = 3.17, *p* = 0.002), the IC group significantly underperformed above and beyond the contribution of intelligence, with a large effect size (*B* = −11.83, *SE* = 3.83, *t* = −3.09, *p* = 0.004; robust Wald (1,43) = 10.05, *p* = 0.002, for the comparison of the fit of the models). Notably, the two groups did not display significant differences in average performance on Phoneme Awareness *(B* = −5.54, *SE* = 4.59, *t* = −1.21, *p* = 0.235) or Pseudoword Repetition (*B* = −8.99, *SE* = 5.01, *t* = −1.79, *p* = 0.0803), although both effects were in the negative direction, consistent with results obtained for other subtests.

Mixed logit modeling of participants’ behavioral accuracy on the experimental task revealed that the BFC group was less accurate in identifying the mismatch in the Initial Phonological Overlap (IPO) condition, compared to the match condition (*B* = −0.70, *SE* = 0.16, *z* = −4.34, *p* = 0.0000143), and was more accurate in identifying the mismatch when the word was neither phonologically nor semantically overlapping or associated with the target (*B* = 2.92, *SE* = 0.71, *z* = 4.11, *p* = 0.0000402). Given that the interactions with the group term for dummy-coded condition effects (i.e., UR and SA effects) both had *z*-values < |0.04| and associated *p*-values > 0.736, we can assume that these effects held in general for the IC group. However, the IC group showed overall reduced accuracy on this task compared to the group of BFC age peers (*B* = −0.46, *SE* = 0.20, *z* = −2.31, *p* = 0.0210). Counterintuitively, however, the IC group also demonstrated a reduced effect of the IPO condition on accuracy (*B* = 0.59, *SE* = 0.23, *z* = 2.55, *p* = 0.0109); i.e., adults in the IC group showed a smaller decrease in accuracy in identifying the match vs identifying the IPO mismatch, compared to the BFC group.

Robust linear mixed modeling of log-transformed reaction times (RT) with random effects of subjects and items on the intercept was performed after excluding incorrect trials and trials with RT > 2500 ms from the analysis. The analysis revealed substantially slower RTs for the IPO and the SA conditions for the combined sample (*B* = 0.17, *SE* = 0.03, *t* = 6.19; and *B* = 0.09, *SE* = 0.03, *t* = 3.17, respectively), indicating that participants took longer to identify mismatch in words that were phonologically or semantically associated with the target word displayed in the picture, than to correctly identify the target. At the same time, the IC group was extra slowed by the IPO condition (for the interaction term, *B* = 0.04, *SE* = 0.02, *t* = 2.22). Coupled with the accuracy findings presented above, this result points to the speed-accuracy tradeoff in processing words that are phonologically consistent with the target for the initial several hundred ms in adults with a history of institutionalization. The IC group was not overall slower than the BFC group, *B* = 0.05, *SE* = 0.05, *t* = 0.95, in responding to the target match condition stimuli.

In sum, the analysis of the behavioral data revealed that Russian-speaking adults with a history of institutionalization display an overall depressed performance across a multitude of language tasks in the standardized testing setting. These deficits are widespread (although sparing phonological working memory and phoneme awareness), large in effect size, and are not explained by group differences in nonverbal cognitive functioning. In addition, in the experimental task, adults in the IC group were overall less accurate in identifying the target word in a cross-modal picture-word matching paradigm, compared to their BFC peers, and displayed a speed-accuracy trade-off when processing distractors for which the acoustic signal significantly overlapped initially with the target.

### Neurophysiological indices of language processing in adults with a history of institutionalization

In this group comparison, we anticipated a diferential profile of ERPs in the two comparison groups. Specifically, based on our previous work, we expected to see a reduced N400 compoent in the Initial Phonological Overlap (IPO) and Unrelated (UR) conditions. Yet, as the ARFA did not register statistically significant group differences in phonological processing, we revisited our expectations *post hoc*, regarding the IPO differences, taking an ambivalent position regarding the IPO condition.

First, we will present the main findings from the ICA decomposition of the event-related average waveforms performed on the combined study sample. Given the limited space, the presentation of the results will be centered around the temporo-spatial factors that a) explain a substantial proportion of the variance in the time-locked average EEG signal; b) differentiate, according to the results of robust linear modeling, the experimental conditions or the two study groups (IC vs. BFC); and c) are interpretable.

At the first step, we performed a temporal independent component analysis with Promax rotation. Model parameters such as factor loadings were estimated using the jack-knife cross-validation procedure. Factor eigenvalues were compared to those obtained using simulated data mirroring the structure of the study data. Using this parallel scree criterion, twenty-four temporal factors were retained and explained 90.78% of the variance in the event-related data. At the second step, factor scores obtained during the first step were further subjected to a spatial Infomax-based ICA. The parallel scree test suggested that five spatial factors should be retained, explaining 71.90% of the variance in the temporal ICA factor scores. The resulting temporo-spatial ICA solution included one hundred-twenty (24 temporal × 5 spatial) temporo-spatial factors. Factor scores were extracted at peak channels and peak time points for those 26 factors that satisfied the minimum criterion of at least 0.05% variance to minimize multiple comparisons by excluding factors that account for mostly noise or subject-specific variance.

**Figure 2:**
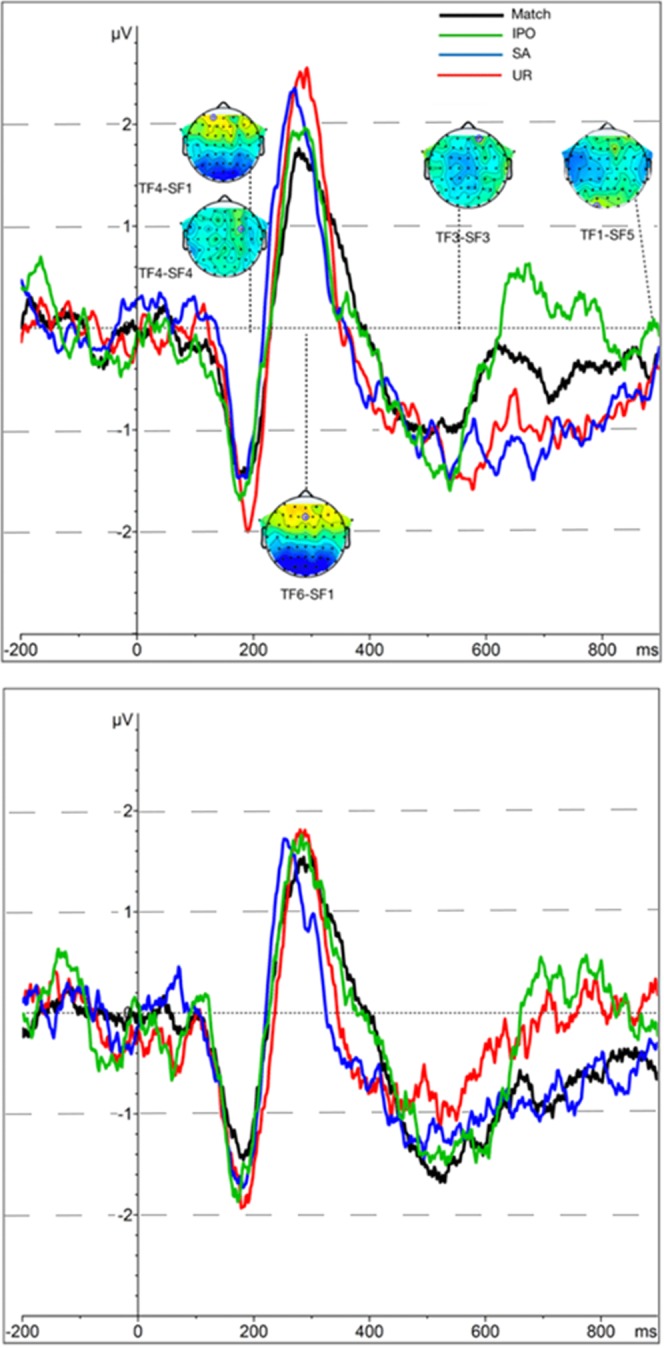
Grand-averaged event-related potential (ERP) waveforms in two groups of participants – adolescents and adults raised by biological families (BFC, top panel) and adolescents and adults raised in institutional settings (IC, bottom panel). TF – temporal factor. SF – spatial factor. IPO – initial phonological overlap. SA – semantically associated. UR – semantically and phonologically unrelated. Scalp maps represent temporospatial factor topographies in their peak channel at their peak latency.

For each of the twenty-six temporospatial ICA factors, a robust linear mixed model that included the random effect of subject on the intercept and age, intelligence, and gender as covariates, was fitted. For five temporo-spatial factors, the value of the *t*-statistic for the fixed effects associated with the group factor exceeded |2.32|. The temporo-spatial factor topographies and the average event-related waveforms are presented in Fig. 2. These five temporo-spatial factors accounted for variation in four time windows. The first two factors, TF4-SF1 and TF4-SF4, both had a peak latency of 188 ms post-stimulus onset, and captured the variation around the first negative peak (N200). For TF4-SF1, the peak was observed in the left-frontal electrodes (i.e., AF3). Although the amplitudes were higher for the UR compared to the match condition, *B* = −0.99, *SE* = 0.33, *t* = −2.95, a significant group by condition interaction, *B* = 1.29, *SE* = 0.48, *t* = 2.67, suggested that the IC group displayed a disproportionately small reduction in amplitude in this condition. This corresponds to the visually identifiable difference in the UR vs. match condition between the groups around the N200 peak. Since this component is thought to be involved in context-modulated anticipation and is sensitive to word expectancy, this result suggests atypically low sensitivity to the low expectancy of the word that is an immediate mismatch (phonologically and semantically) of the target depicted in the picture. At the same time, a negative temporo-spatial factor TF4-SF4 (peaking at TP7) displayed sensitivity to IC vs BFC group status in the baseline condition when the presented auditory word matched the picture: adults with IC experience displayed significantly smaller TF4-SF4, *B* = −1.72, *SE* = 0.65, *t* = −2.65, compared to their BFC peers.

TF6-SF1 represented the frontal positivity – parietal negativity complex (P300-N400) with a 242 ms post-stimulus onset. For this temporo-spatial factor, several effects were observed. First, amplitudes were more negative in the Semantically Associated, SA, (*B* = −1.02, *SE* = 0.28, *t* = −3.64) and, tentatively, in the UR (at *B* = −0.57, *SE* = 0.28, *t* = −2.03) conditions, compared to the match condition. At the same time, the IC group demonstrated markedly more negative amplitudes than the BFC group in the referent match condition (*B* = −1.38, *SE* = 0.35, *t* = −3.79). Significant two-way interactions between group and the SA condition code (*B* = 1.05, *SE* = 0.40, *t* = 2.63), and between group and the UR code (*B* = 1.18, *SE* = 0.40, *t* = 2.95), were also established. Together, these findings suggest that for the BFC group, the P300/N400-like TF6-SF1 temporo-spatial factor was sensitive to the mismatch, whereas in the IC group it was not. This finding is consistent with accounts that implicate the P300-like response in attention allocation when processing novel or surprising stimuli. At the same time, in our data, this positivity was accompanied by broad parietal negativity (frequently seen in speech event-related data in the context of expectation violation) that is thought to reflect processes of lexical selection in situations when contextual expectations (e.g., generated by the picture) do not support the form-based activation of the lexical candidate (e.g., consistent with the auditory form). In this case, the more negative amplitudes of the N400-like negativity reflect that the pool of activated candidate words in the SA and UR conditions do not contain semantic features that fit the concept depicted in the picture. Note that although the reduction of the N400 would be expected in this case for the SA condition, the stimuli in our experiment were semantically associated (i.e., co-occurring) rather than related in terms of overlapping feature sets.

Finally, TF3-SF3 represented the negative-polarity factor with a fronto-central distribution that peaked at 518 at Cz. The component likely represented the processes responsible for the identification of lexical and phonological mismatch (that comes in later in the IPO condition due to the temporal overlap with the match word) and corresponded to mismatch negativity that temporally overlapped with the N400 time window. Consistent with this interpretation, the BFC group tended to display more negative amplitudes in the IPO compared to the match condition (*B* = −0.70, *SE* = 0.32, *t* = −2.21); this effect was essentially absent in the IC group, as demonstrated by the significant group by IPO condition term interaction, *B* = 1.09, *SE* = 0.45, *t* = 2.40. Although the last temporo-spatial factor, TF1-SF5, also displayed a number of effects, the factor peaked at 898 ms, right at the edge of the analyzed segments, and therefore was not interpreted.

In sum, our study demonstrated that adults with a history of institutionalization displayed reduced neural sensitivity to semantic and phonological mismatches in a cross-modal picture-word matching paradigm. Preliminary analyses limited to the IC group did not reveal significant pairwise correlations between behavioral language development scores and neurophysiological indices of lexical processing, or between these variables and length of institutionalization. Note, however, that these analyses inherently had low power to detect even moderate effects since they were limited to n = 24.

### Predicting IC vs. BFC group membership using intelligence, language, and ERP measures

**Figure 3:**
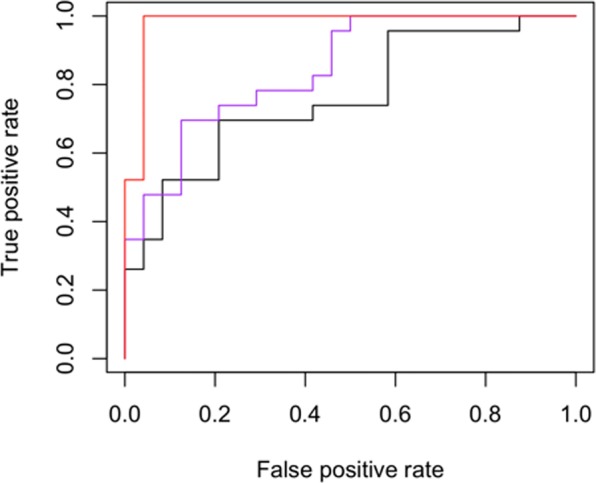
Receiving Operator Characteristic (ROC) curves for quadratic discriminant models predicting group status. Black = model with only demographic variables and nonverbal intelligence; purple = model with ARFA scores added; red = model with temporospatial ICA scores added.

In order to predict IC vs. BFC group membership, three quadratic discriminant analysis (QDA) models were fit. The first model only included the demographic variables and nonverbal intelligence. The model showed 52% sensitivity (12/23 IC actual/predicted) and 83% specificity (20/24 BFC actual/predicted). Adding the general language development score derived from ARFA improved the model accuracy to reach 61% sensitivity (14/23) and 88% specificity (21/24). Adding the factor scores for the temporo-spatial factors presented in the previous subsection improved the model accuracy further, dramatically increasing sensitivity (87% or 20/23) with retained specificity (88% or 21/24). Receiving operator characteristic (ROC) curves are presented in Fig. 3.

The results of discriminant modeling suggest that language has unique discriminating power with respect to identifying individuals with IC experience in our sample. Moreover, our neurophysiological measures captured additional sources of variation and dramatically improved the model sensitivity. Importantly, it resolved the inconsistency between the behavioral (3.1) and ERP (3.2) results; although not all subtests of the ARFA demonstrated statistically significant group differences, and although, behaviorally, there appear to be some compensation of language-based differences between the two groups, the residual differences in language processing in the brain add precision (26%) to differentiating the IC and BCF groups.

## Discussion

Language is widely recognized as a unique species-specific ability that offers highly specialized representational capacity and regulatory potential but also has computational requirements. The postulated unique status of language, the presence of sensitive periods for speech and language development, and recent findings that link children’s language development to the properties of the information afforded by their environments together highlight the importance of input and its richness as foundational data for uptake. Motivated by findings of atypical cognitive development and delayed language development in special vulnerable populations of children raised in institutional settings, we examined behavioral and neurophysiological indices of language development in a sample of Russian-speaking adolescents and adults with and without a history of IC.

Behavioral assessment data revealed that adults with a history of institutionalization underperformed on most study measures. Consistent with the prior literature on the general cognitive functioning of children raised in institutions, we found that adults with a history of institutionalization had markedly lower nonverbal intelligence scores than their age peers raised by biological families. These differences are noteworthy as they effectively illustrate the long-term effects of the impoverished environment and psychosocial deprivation that characterize institutionalization on cognitive development. Critically, these differences did not account for the profound deficit in language development we observed in the group of adults with a history of institutionalization. These deficits were large in effect size (almost 1 SD in excess of deficits that would be predicted from nonverbal intelligence scores alone) and affected core language domains, including lexical development, morphosyntax, and literacy skills. The presence of these differences is consistent with studies on the development of language in institutionalized children reviewed in the Introduction, and points to the insufficiency of compensatory developmental processes in recovering from the possible early “insult” (in terms of deprivation of input) to the developing system that might be the beginning of a cascade of developmental deficits accumulating over time.

Although early institutional care in the Russian Federation has been described as adequate with regard to such biological needs of children as proper nutrition and medical care, it has also been characterized as being psychosocially depriving^9,69^. In orphanages and baby homes, children typically live in wards of 7–8 children each, grouped by age and medical diagnosis, and are cared for by multiple caregivers working in rotating shifts. Such conditions are associated with attachment disturbances, lack of caregiver responsiveness, low stability of the environment, and limited amount of child-directed interactions^9^. All of these factors likely affect key parameters of the linguistic input that a child receives, as well as the ways in which that child interacts with that input.

Speculatively, language delays in children raised in institutional settings are likely largely attributable to early lexical development, when rapid growth is expected and is in fact predictive of future language outcomes in other domains, including syntax and written language development^41,50,70,71^. When input is suboptimal, language delays become language deficits due to the presence of sensitive periods, since early lexical development effectively bootstraps the acquisition of syntax, and both its breadth (size) and depth (featural and contextual specification) alter the processing dynamics realized by the developing linguistic system.

To the best of our knowledge, our study is the first to document atypical neural dynamics of spoken language processing in adults with a history of institutionalization. Using a high-density EEG setup, we performed an ICA-based decomposition of event-related waveforms that identified temporo-spatial factors corresponding to several components. Adults with a history of institutionalization displayed atypical responses to violations of lexical expectation generated in a cross-modal picture-word paradigm. Specifically, unlike their peers raised in biological families, adults with a history of institutionalization did not demonstrate a reduction in the N200-like factor amplitude in response to pictures semantically and phonologically unrelated to the target word, nor in the modulation of the P300/N400-like factor amplitude. These findings suggest that IC has lasting detrimental effects on individuals’ language development, detectable at the neural level during online spoken word processing. The precise nature of these deficits is unknown, however, our results suggest that the dynamics of lexical activation in this population are characterized by decreased sensitivity to lexical mismatch, whether it occurs at the beginning or in the middle of the word. The affected components span multiple hypothetical mechanisms, from phonological processing^72–74^ to early semantic processing and early lexical selection^75^. Consistent with findings from studies of children at risk for language difficulties^50^, we found that adults with history of IC show atypical N400-like amplitudes, possibly because of language deficits due to unstable or underspecified lexical representations, as the component is thought to index stimulus-induced semantic activity that occurs against the background of both long-term knowledge and recent experiences (e.g., experimentally generated top-down expectations) that influence the activation of lexical and semantic categories^76,77^.

The altered dynamics of neural activation during word processing in the IC group point to lexical development as a particularly vulnerable language domain. Yet, in our sample, behavioral language deficits did not correlate significantly with the ERPs. We would like to suggest that this result points to the importance of focusing on neurally-based measures of cognitive processes in older populations for the purpose of capturing unique variance in their developmental outcomes. Indeed, the results of our discriminant analysis suggest that neural measures of language development substantially improved the model’s classification accuracy above and beyond behavioral measures of intelligence as well as language.

Our study has several limitations that are important to consider when interpreting the results. First, our inferences about long-term outcomes in Russian-speaking adults with a history of institutionalization are based on the results of a cross-sectional, rather than prospective longitudinal study, thereby rendering claims about developmental dynamics speculative. Partial support from these results comes from our recent study of children between the ages of 1 year and 4 years raised in Russian orphanages that documented substantial lexical deficits, as well revealing altered neural dynamics of lexical processing^49^. Second, given that SES is not only subject to assortative mating but also has a substantial genetic component, it is possible that developmental deficits in our sample (and children abandoned to orphanages in general) stem from genetic factors shared between intelligence and SES^78^. Importantly, however, the results of our study suggest that language difficulties observed in adults with a history of institutionalization exceed their nonverbal deficits, pointing to the possible accumulation of weaknesses that is exacerbated by impoverished input. Third, although we did control for nonverbal intelligence, it is possible that in the domain of nonverbal cognition, behavioral and neural indices of development can have unique predictive power, and future studies should examine the extent to which differences in functional activation during language processing can be attributable to deficits in general neural efficiency.

The results of our study add to the growing body of literature that highlights language as a system that is sensitive to deficits in the quantity and quality of input and early caregiving environments, and that implicates institutionalization’s association with poor long-term language outcomes both at the level of brain and behavior. The field will undoubtedly benefit from a systematic interrogation of the linguistic environments children are exposed to in institutionalized settings, and the proper modeling of the relationships between these properties and children’s concurrent and prospective language development, controlling and accounting for individual differences in other cognitive domains. Such studies may result in effective early intervention recommendations, as well as both the recognition of and recommendations for support of struggling adult learners whose developmental trajectories have been altered by impoverished environments.

## Data Availability

Behavioral and neurophysiological data are available from the authors upon request.
